# Effects of Ultraviolet-B Irradiance on Intraspecific Competition and Facilitation of Plants: Self-Thinning, Size Inequality, and Phenotypic Plasticity

**DOI:** 10.1371/journal.pone.0050822

**Published:** 2012-11-30

**Authors:** Rui-Chang Zhang, Yue Lin, Ming Yue, Qian Li, Xiao-Fei Zhang, Xiao Liu, Hong Chi, Yong-Fu Chai, Mao Wang

**Affiliations:** 1 Key Laboratory of Resource Biology and Biotechnology in Western China (Northwest University), Ministry of Education, Xi’an, China; 2 Institute of Forest Growth and Computer Science, Dresden University of Technology, Tharandt, Germany; 3 Helmholtz Centre for Environmental Research - UFZ, Department of Ecological Modelling, Leipzig, Germany; RIKEN Plant Science Center, Japan

## Abstract

(1) The effects of facilitation on the structure and dynamics of plant populations have not been studied so widely as competition. The UV-B radiation, as a typical environmental factor causing stress, may result in direct stress and facilitation. (2) The effects of UV-B radiation on intraspecific competition and facilitation were investigated based on the following three predictions on self-thinning, size inequality, and phenotypic plasticity: i) Self-thinning is the reduction in density that results from the increase in the mean biomass of individuals in crowded populations, and is driven by competition. In this study, the mortality rate of the population is predicted to decrease from UV-B irradiance. ii) The size inequality of a population increases with competition intensity because larger individuals receive a disproportionate share of resources, thereby leaving limited resources for smaller individuals. The second hypothesis assumes that direct stress decreases the size inequality of the population. iii) Phenotypic plasticity is the ability to alter one’s morphology in response to environmental changes. The third hypothesis assumes that certain morphological indices can change among the trade-offs between competition, facilitation, and stress. These predictions were tested by conducting a field pot experiment using mung beans, and were supported by the following results: (3) UV-B radiation increased the survival rate of the population at the end of self-thinning. However, this result was mainly due to direct stress rather than facilitation. (4) Just as competitor, facilitation was also asymmetric. It increased the size inequality of populations during self-thinning, whereas stress decreased the size inequality. (5) Direct stress and facilitation influence plants differently on various scales. Stress inhibited plant growth, whereas facilitation showed the opposite on an individual scale. Stress increased survival rate, whereas facilitation increased individual variability on the population scale. (6) Trade-offs between competitions, facilitation, and direct stress varied in different growing stages.

## Introduction

The ultraviolet irradiance (280 nm to 320 nm) reaching the Earth’s surface has significantly increased since 1979 due to the unprecedented destruction of the ozone layer. Recent studies predict further increase of ultraviolet irradiance in the future [1], [2]. Several studies relative to the impact of enhanced UV-B radiation on plant physiology, morphology, growth, and development have been conducted [3], [4], [5], [6], [7], [8]. The immediate effects of increased UV-B in biotic interactions among plant individuals have great potential consequences [9], [10]. Studies showed that enhanced UV-B radiation, as an abiotic stress factor, alter the competitive interactions among plants both at intra- and interspecific levels [3], [11], [12], [13]. Thus, UV-B radiation greatly affects the plant population and community structure [14], [15]. Nevertheless, the potential role of positive interaction among plants under the stress of enhanced UV-B radiation, have not been investigated.

Negative interactions, such as competitions, are dominant biotic factors that shape plant populations and communities [16]. The important role of positive interactions (facilitation) in the structure of plant system has been proven by several studies [17], [18], [19]. Plant facilitation is defined as the beneficial effects of neighbors through the amelioration of habitat (e.g. moderation of stress), which is particularly important when considering the performance of plants under environmental severity (i.e. abiotic stress) [20]. The stress-gradient hypothesis (SGH) [21] predicts the a shift from competition to facilitation along environment gradients, wherein facilitation should be dominant over competition in the development of population and community structure under high-stress conditions [22], [23], [24]. This theory was supported by several studies [25], [26], [27], [28] and these studies also proposed an idea that can expand the conceptual framework in ways of explicitly considering the type of stress gradient and the characteristics of life-history strategy [29]. In connection with intraspecific competition, the categories of stress have become more important because individuals of certain species have the same life-history strategy of CSR [30], [31]. Although SGH was originally formulated at the interspecific level, its validity at the intraspecific level was rarely tested.

The effects of enhanced UV-B radiation on plants would have two aspects if facilitation appears. Direct stress inhibits plant growth and facilitation, which is beneficial to individuals in the population. However, as a non-resource-related abiotic stress, enhanced UV-B radiation is different from resource-related stress, such as water, light, and nutrients. The facilitation introduced by UV-B radiation mainly reduces the damage from UV-B irradiance rather than improve the utilization of resources. If facilitation does exist in the context of UV-B radiation, this study proposes three predictions about size inequality, self-thinning, and phenotypic plasticity. These predictions will be verified by applying the SGH theory in a field pot experiment.

The mortality rate of plants in a population increases as the population density increases, which is known as density-dependent mortality or self-thinning. Self-thinning is caused by competition. Self-thinning occurs when crowding is severe, is concentrated among the smallest individuals, and accordingly alter the size distribution [32]. Nonetheless, plants growing with supplementary UV-B radiation may have a different mortality rate. The first prediction describes that mortality rate is decreased by UV-B radiation. This prediction is based on facilitation and direct stress. First, plants receive more benefits from their neighbors as the intensity of UV-B radiation increase in light of stress-gradient hypothesis. Furthermore, neighboring plants protect individuals from UV-B radiation damages because of facilitation. Hence, the survival rate of population under high UV-B irradiance level is higher than those under low UV-B irradiance level. This explanation implies that the enhanced UV-B radiation, and not self-thinning, was the primary cause of death in individuals. Second, direct stress delays self-thinning and reduces the mortality rate of the population by reducing the growth rates of individuals. Hence, the survival rate of plants was higher under enhanced UV-B radiation compared with the ambient group. The second reason is based on the premise that self-thinning was the main reason for plant death.

Competition among individuals usually increases size variation within plant populations. Previous studies concluded that the major factor that generates size variation in crowded plant populations is size-asymmetric competition (i.e. larger plants have more advantages due to their relative size in competition compared with smaller ones). Larger individuals with more advantages become gradually dominant in the population. Accordingly, size inequality increases with density. The observed increase in size inequality of populations supports the hypothesis of size-asymmetric competition [32], [33]. The effects of self-thinning should also not be neglected because size inequality keeps increasing until the onset of self-thinning as a crowded population develops. However, this situation may change if the population is placed under enhanced UV-B radiation. The second prediction describes that direct UV-B stress decreases the size inequality of the population because abiotic stress decreases the growth rate of all the individuals, but not a part of them. Nevertheless, the effects of facilitation on size inequality are undetermined. There are two possibilities regarding this condition. Larger individuals are more damaged from UV-B stress than the smaller individuals. Consequently, facilitation decreases the size inequality. In the second case, larger individuals have more benefit from their neighbors because they have more interactive area than smaller ones. Therefore, size inequality of the population is increased by facilitation, indicating that facilitation is unsymmetrical.

The third prediction describes that plant phenotypic plasticity changes in different stages under enhanced UV-B radiation. Plant phenotypic plasticity is caused by the changes in the allocation of resources in the plasticity of plant size and morphology [34], [35]. Phenotypic plasticity enables plants to adapt to different environments. Plant height can be special in all the morphological indices because of the important trade-off between obtaining light resources and avoiding damages for plants growing under enhanced UV-B radiation. Plants are apt to be taller to struggle for light resources at higher densities, whereas they tend to be shorter to avoid the damages of supplementary UV-B radiation [36]. Moreover, we speculate that the strategy for resources and stress should be changed in different growth periods because the impact of competition and UV-B radiation is not alterable under different growing stages.

The current study aims to test the three hypotheses on plant population and address the following problems: (1) Identify the main factor that decreases the mortality rate of the population during self-thinning, direct stress, or positive interactions; (2) Determine whether facilitation increases or decreases size inequality of population; and (3) Distinguish the mechanism in the shift in strategy on obtaining light resources and avoiding stress under different growth periods.

## Results

### Self-thinning

**Figure 1:**
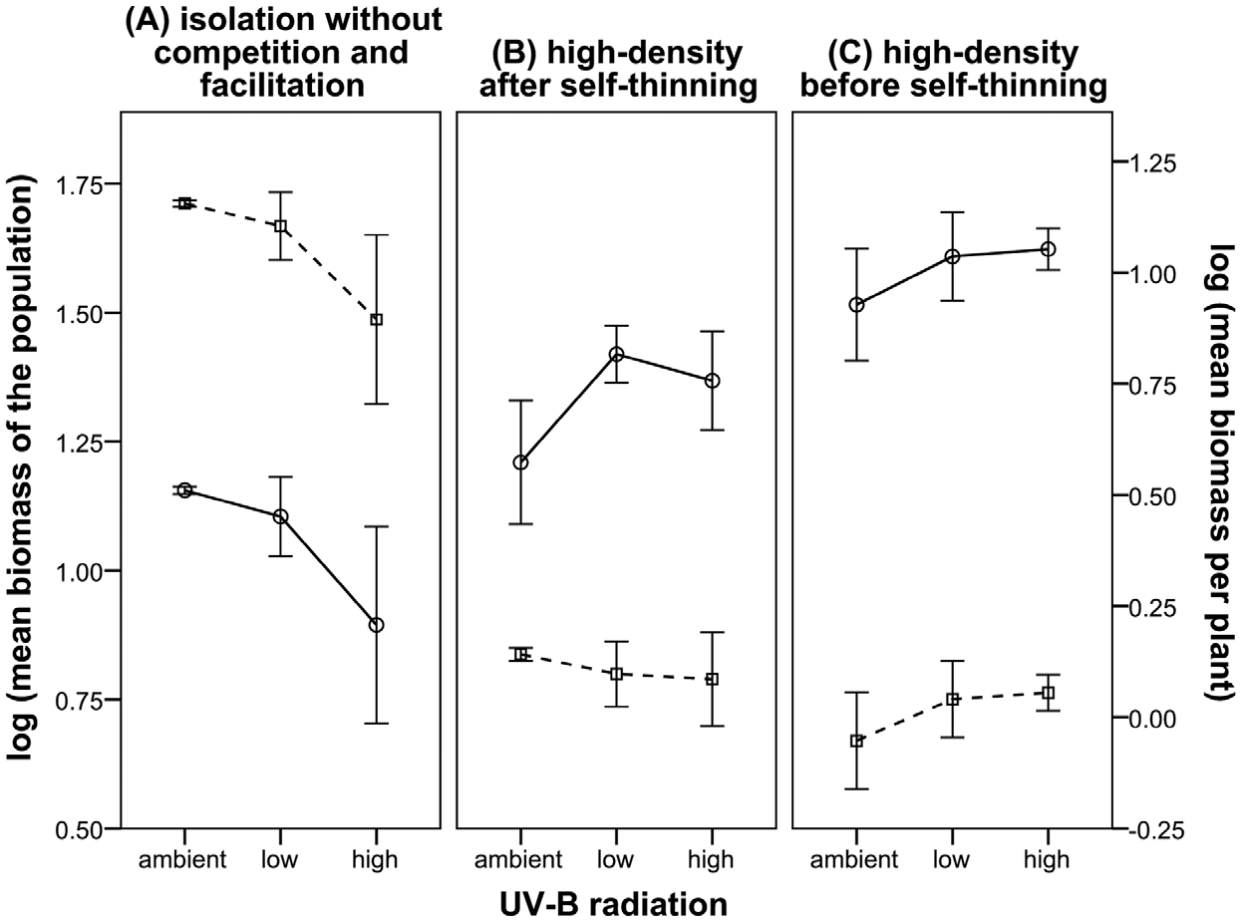
Total and mean biomass of mung beans under different levels of UV-B radiation and density. Plants were grown for 86 days (three months) under three density levels [1 plant in a pot (isolation), 7 plants in a pot (low-density)] and 37 plants in a pot (high-density)]. The plants were exposed to the following three UV-B radiation levels: 8.05 kJ·m^2^ UV-B_BE_ (ambient), 8.05+3.60 kJ·m^2^ UV-B_BE_ (low-enhanced), and 8.05+5.62 kJ·m^2^ UV-B_BE_ (high-enhanced). There were three replicates for each treatment. The plants were sampled at the end of each month during the whole growing season. The solid line represents the total biomass of the mung bean population, whereas the dotted lines represent the mean biomass. Panels A and B show the last month’s results (strongly self-thinning was observed) and panel C shows the second month’s trends. Data are means ± SD (there are 9 pots for each panel; n = 9 plants in panel A, n = 157 plants in panel B and 333 plants in panel C).

**Figure 2:**
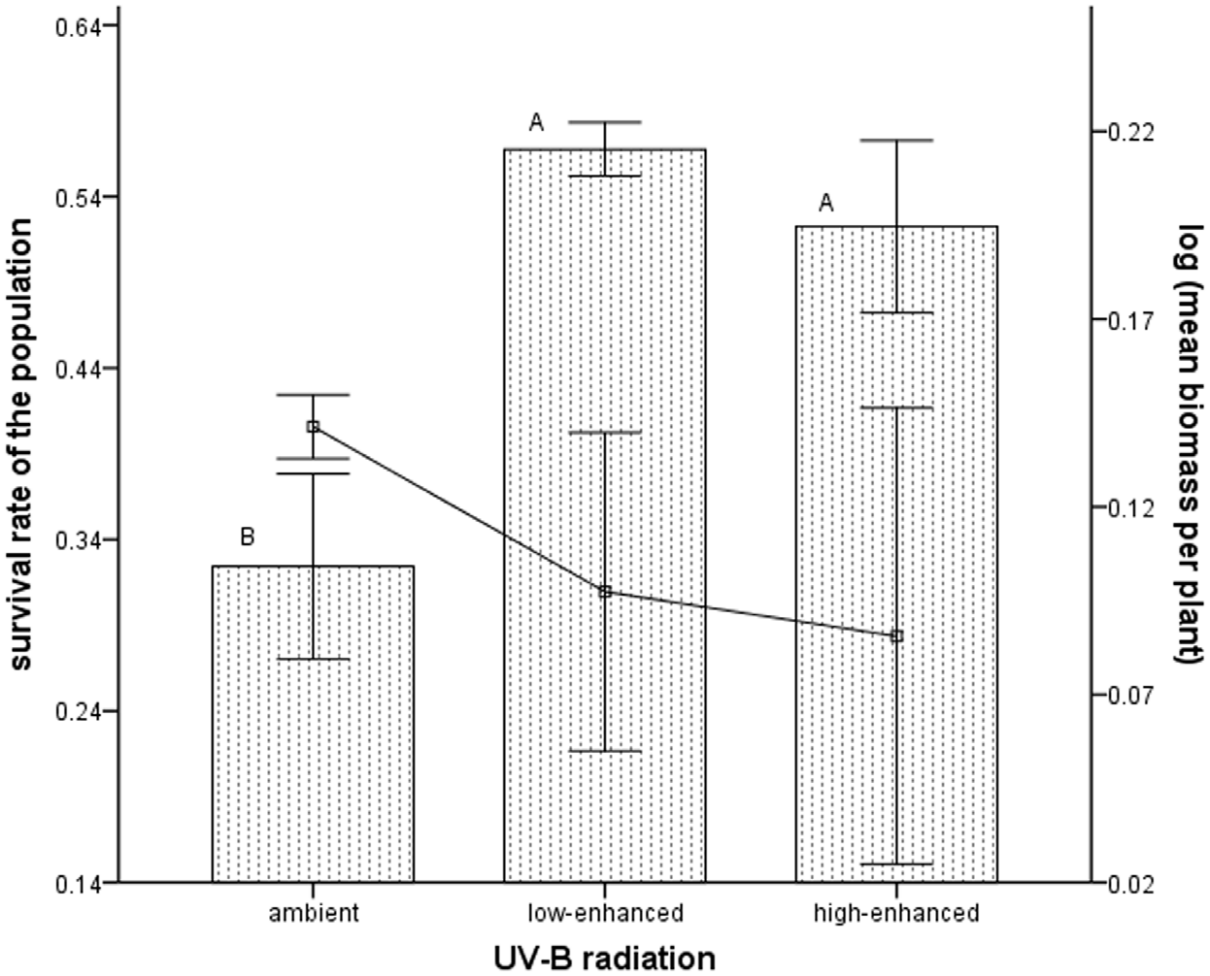
Survival rate and mean biomass under different levels of UV-B radiation in the last stage. The high-density group under different UV-B radiation levels (ambient, low-enhanced and high-enhanced) showed the relationships between the survival rate (bar) of mung bean population and the mean biomass of individuals of the population (solid line) in the last month during self-thinning. The bars surmounted by different letters are significantly different (P < 0.05) according to Duncan’s test and data are means ± SD (there are 9 pots and n = 157 plants).

The biomass of plants in the isolated group declined with UV-B radiation (Figure 1A), indicating that direct stress decreases the biomass. Strong self-thinning was observed in stage 3 (Figure 2). Meanwhile, the mean biomass declined with UV-B radiation (Figure 1B), implying that the biomass of plants was decreased by the net effects of stress and facilitation in stage 3. However, both total biomass of the population and the mean biomass per plant increased with UV-B radiation at high density in stage 2 (Figure 1C), suggesting that facilitation took place in stage 2. Therefore, facilitation increased individual biomass. The results in stage 2 show that the total biomass of the population under the ambient UV-B radiation was still lower than the low-enhanced radiation and high-enhanced UV-B radiation groups although the effects of UV-B stress were higher than the facilitation in stage 3 (Figure 1B). This condition was due to the least number of survival rates among individuals under ambient UV-B.

Populations that experienced UV-B stress and facilitation had higher survival rates than those under normal circumstance (Figure 2). However, no statistical difference was observed between populations under low- enhanced UV-B radiation and those under high-enhanced UV-B radiation.

### Size Inequality

**Figure 3:**
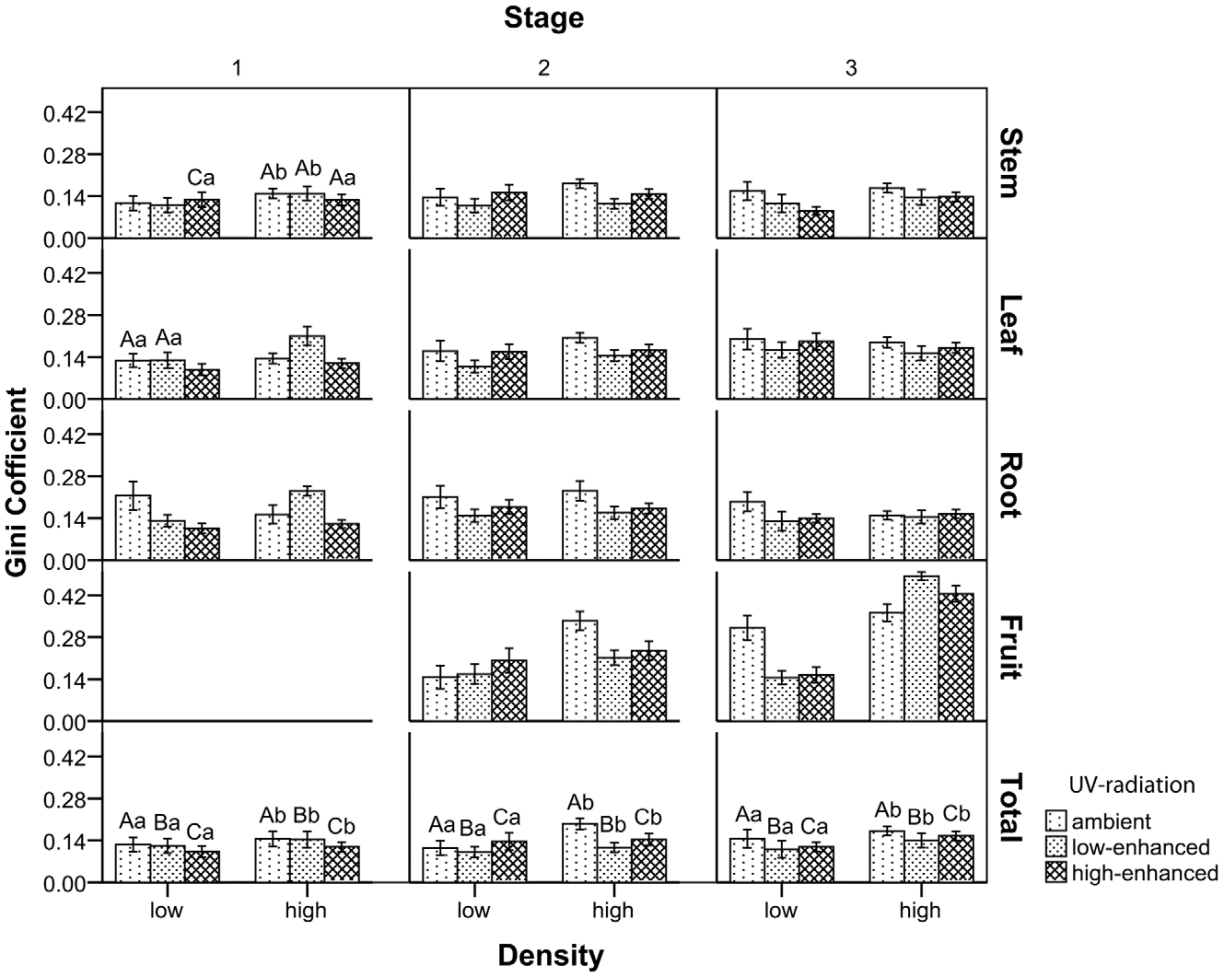
Effects of enhanced UV-B radiation and competition on the Gini Coefficient of biomass at three times. The Gini coefficient of the stem, leaf, root, fruit, and the whole plant biomass were calculated after the plants were sampled in each month. Only the low-density and high-density groups under each UV-B radiation level were calculated because there was only one plant in each pot for the isolated group. Ninety-five percent confidence intervals for unbiased values were determined using the bootstrapping technique (5000 samples). The same capital letter indicates no significant difference at 0.001 level among different radiations in the same parameters and same density of mung beans. The same small letter indicates no significant difference at 0.001 level among various densities at the same parameters and same radiation of mung beans. The Duncan's multiple range test calculated the significance at p <0.001 level. The unmarked bars followed the total group, and data are means ± SD.

The Gini Coefficient in isolation was not calculated because there was only one individual in one pot. Table S1 shows that in the three stages, the Gini Coefficient of these plants were all significantly affected by the UV-B treatments, density levels, and two-way interaction between UV-B×Den (density) (P<0.001). The conduction of multiple comparisons was difficult and unpractical in the two-way ANOVA because of the interactions between UV-B radiation and density. Thus, in this experiment, one factor had to be fixed before the multiple comparisons. Results showed that there was no consistent trend in the Gini Coefficient of each plant part (root, stem, leaf, and fruit) during the three stages (Figure 3). However, coincidental patterns at different radiation and density levels were observed upon focusing on the total plant biomass.

**Figure 4:**
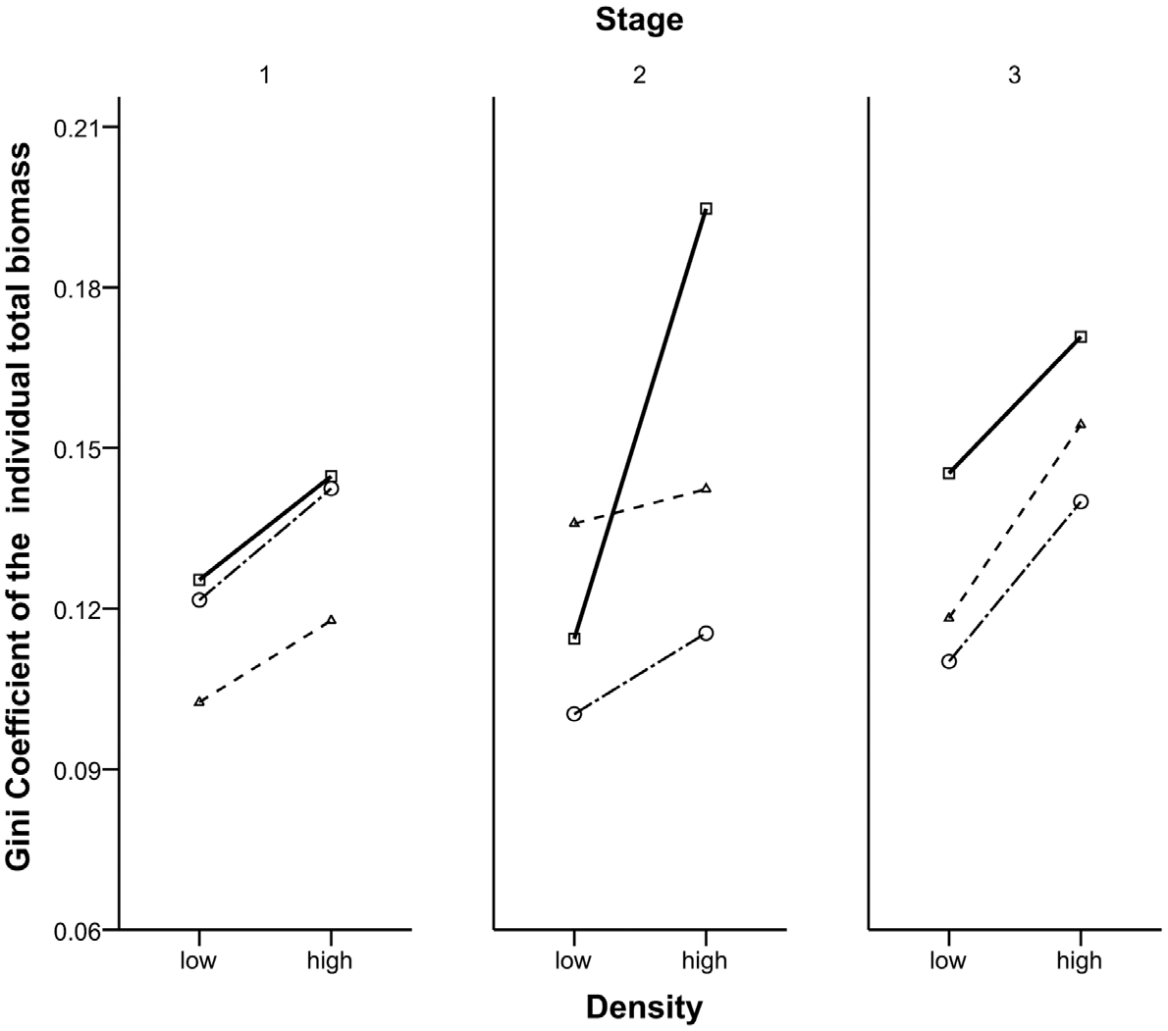
Trends of Gini Coefficient under different UV-B and density levels in the whole growing season. The Gini Coefficient of the individual total biomass of low- and high- density groups under ambient conditions (-□-), low-enhanced UV-B radiation (-○-), and high-enhanced UV-B radiation (-△-) was calculated respectively in each month after the plants were sampled. Bootstrapping determined the confidence intervals (95%). Each data point represents the result calculated from 5000 samples based on bootstrapping. Otherwise, as for Figure 3.

The enhanced UV-B radiation, which represented both UV-B stress and facilitation, strongly influenced the size inequality of the population with competition. The competition increased the size inequality (Figure 4), regardless of whether there was UV-B stress and facilitation. Furthermore, the minimum Gini coefficient was observed at low density and the maximum Gini coefficient was found at high-density in the field pot experiment (Figure 3).

The impacts of enhanced UV-B radiation on size inequality were not as simple as competition; they were related to the growing stage of the plant populations (Figure 4). The population had lower size inequality under normal environmental condition than that under both low-enhanced and high-enhanced UV-B radiation in all three stages. However, the high-enhanced group had the highest size inequality in stage 2 when the density was low. Moreover, size inequality of the populations under low-enhanced UV-B radiation was higher than that under high-enhanced UV-B radiation stage 1. However, it was the lowest in the last two stages (Figure 4).

### Phenotypic Plasticity

**Figure 5:**
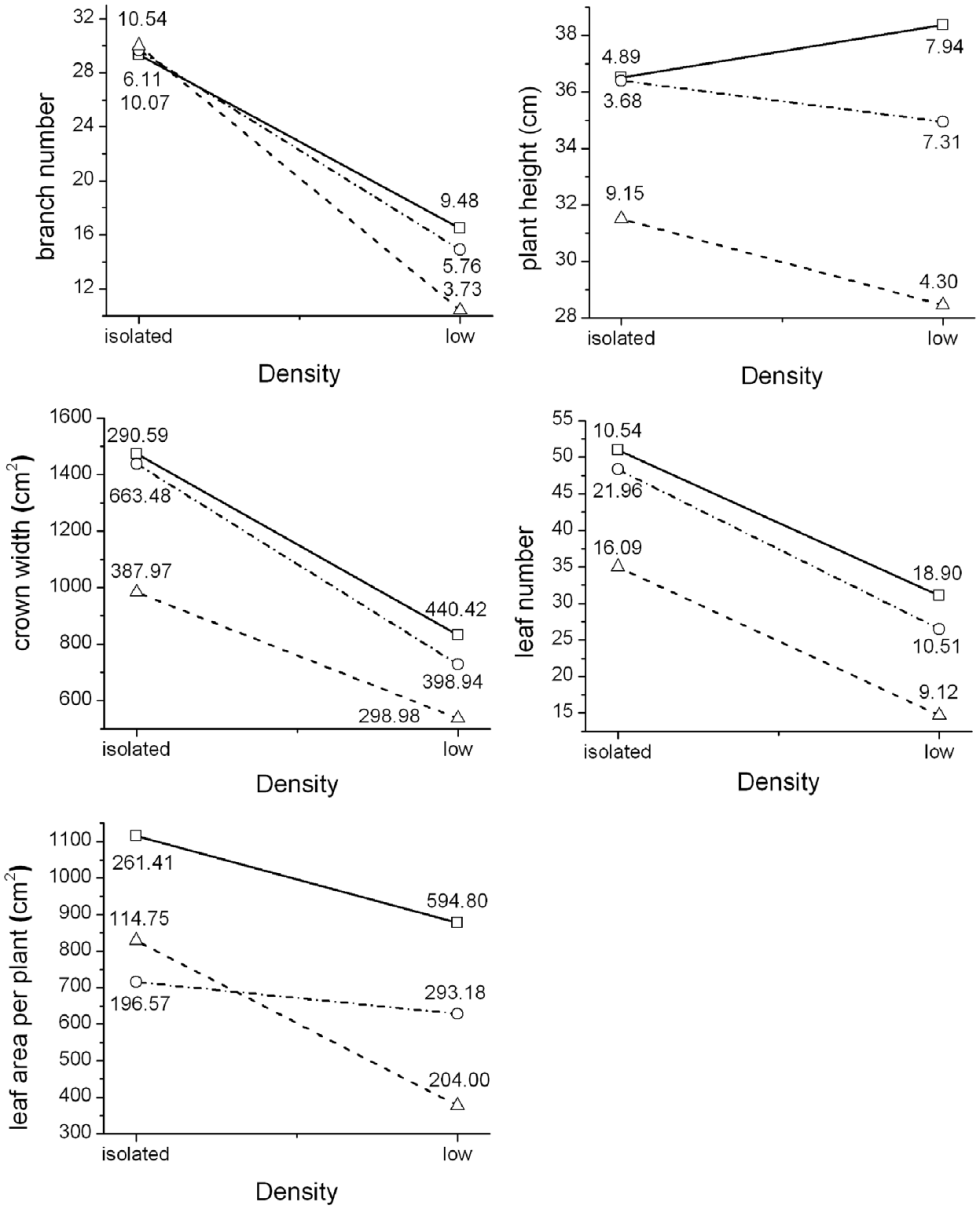
Morphological indices at different levels of density and UV-B radiation in last stage. Isolated and low-density groups under each UV-B radiation level measured the five morphological indices of mung beans in every month before biomass was measured. The morphological indices were branch number, plant height, leaf number, crown width, leaf area per plant. This figure only shows the results in the last month. The three symbols represent ambient conditions (-□-), low-enhanced UV-B radiation (-○-), and high-enhanced UV-B radiation (-△-). The number next to each point indicates the SD. In the last month, the morphology of eleven plants (low-density group) was destroyed. Thus, there were n = 52 plants for the low-density group and n = 9 plants for the isolated group.

Except for branch number and plant height, all morphological indices (leaf number, crown width, and leaf area per plant) decreased with the increase in density and UV-B radiation (Figure 5). The branch number increased as UV-B radiation increased in isolation. Plant height increased with density under ambient condition, whereas plant height decreased with density under both low- and high- enhanced UV-B radiations.

Plant height showed different trends in different stages. UV-B radiation decreased plant height, but was increased with density under ambient condition in all three stages. In stage 1, the height increased with density under low- and high-enhanced UV-B radiation; however, the result was totally opposite in the last stage. Moreover, plant height decreased with density under high-enhanced UV-B radiation, but remained stable under low-enhanced UV-B radiation in stage 2.

## Discussion

In this study, the effects of direct stress and facilitation on populations with and without mortality were investigated. In the field pot experiment, the effects of direct stress and facilitation were too difficult to separate due to the close link between abiotic stress and positive interactions. Therefore, the following assumptions were made:

(1) Facilitation only takes place when stress is present. Facilitation increases with abiotic stress based on the current study on facilitation. Positive interactions help individuals to ameliorate the undesirable conditions caused by stress. Positive interaction also protect plants from the damages of stress and accelerates the growth of individuals in the population (2) Every individual obtains resources from an area around them, whereas neighboring plants assist and compete with each other where their areas overlap [37]. However, the overlapping area is relatively small in the early phases of growing season. Hence, the interactions, such as competition and facilitation among individuals, were assumed to have minor effects in the first stage; (3) Dead plants were assumed to neither facilitate nor compete with other individuals. Thus, dead plant should be excluded from the analysis for the simplicity in interpreting and analyzing the results [32].

### Self-thinning

Our results from the field pot experiment supported the previous prediction that enhanced UV-B radiation can reduce the mortality rate of individuals during self -thinning.

First, competition decreased the mean biomass of plants (Figure 1A and Figure 1B). Second, direct stress decreased the mean biomass. However, facilitation increased the individual biomass. Furthermore, survival rate decreased rather than increased with mean biomass of individuals. Based on these results, we can conclude that stress, instead of positive interactions, increased the survival rate of population. The balance of competition, stress without facilitation (both decreasing mean biomass), and positive interactions (increasing mean biomass) determined the mean biomass per plant. In terms of the whole growing season, the effects of UV-B stress and competition on mean biomass were more than the positive interactions (Figure 1B).

Based on density-dependent mortality, direct stress postponed the inception and lessened the rate of plant growth by reducing growth rates. Thus, the number of survivors at the end of the growing season was lower under stressful environment than that under normal environment. This finding was consistent with previous results from various studies, such as the field pot experiment on *Triticum aestivum* under drought stress [38] and the zone-of-influence [37] based on the WBE model [39]. These studies demonstrated that mortality rate is interrelated with the rate of growth in self-thinning.

Interestingly, stress influences plants differently on various scales. On the individual scale, the direct effects of stress on plants, which inhibit plant growth, is negative for isolated individuals. However, it can also increase the survival rate of plants on the population scale, which is positive for a group of individuals.

### Size Inequality

The enhanced UV-B radiation decreased the size inequality of the population in all growing stages. However, the trend was not simply monotonic, except for stage 1. Size inequality initially decreased, and then increased with UV-B radiation in stages 2 and 3. However, size inequality decreased monotonically with stress in stage 1. The death of plants in stage 3 complicated the analysis of plant size inequality because it decreased the size inequality of survivals during self-thinning and these survivors were quite equal in size after the extensive mortality [32], [40]. Still, a non-monotonic decrease in the relationship of size inequality and UV-B irradiance was observed in the last stage. Nonetheless, plant populations under ambient condition showed less survival rates than populations under low- and high-enhanced UV-B radiation.

Consequently, according to assumption (2), direct stress decreases the size inequality of populations simultaneously when facilitation increases size inequality. Otherwise, we are unable to interpret the monotonic decrease in the relationship of size inequality and UV-B irradiance in the early stage and the non-monotonic decrease in the relationship during the last two stages. In addition, the balance among positive interactions (increasing variation), stress, and mortality driven by competition (both decreasing variation) determined the size inequality of the populations [37].

As a result, the second explanation is more reasonable. Early in the stand development, the spatial pattern was more important than the symmetry of competition within the population. However, the size asymmetry of competition became more important after the plants grew and the competition intensified [32]. In the beginning of the field pot experiment, individuals grow in a uniform spatial pattern. However, soil conditions and other factors are not exactly the same for each individual, thereby resulting in size variation. Larger individuals tend to share more damage from supplementary UV-B radiation than the smaller ones because of their larger size. However, larger individuals also benefit from positive interactions more greatly than smaller ones because of their larger overlapping area. Some individuals are facilitated by neighbors, whereas some are even not. Nevertheless, larger individuals obtain more benefits than damages. Thus, the overall inequality of size in the population increased at the end. This result was supported by the experiments in this study (Figure 4).

Therefore, just as kin recognition [41], [42], positive interactions are also sometimes linked to altruism. Nonetheless, the trade-off between competition and facilitation may not be purely altruistic for whole individuals. Positive interactions are also asymmetric exactly as competition in the population due to the more reward than expense for larger ones.

Furthermore, the influence of facilitation on different scales also differs. Facilitation increases the biomass of individuals on individual scale positively, whereas the individual variability is increased by facilitation on population scale. On the contrary, direct stress affects plants negatively on the individual scale, but it decreases size inequality on population scale.

### Phenotypic Plasticity

In addition to positive interactions and competition, trade-off between light resources and damages from UV-B radiation are also important in the development of plants. UV-B radiation decreased the leaf numbers, crown width, leaf area, and height of plants. However, UV-B radiation slightly increased the branch number at lower density [4], [43], [44]. Nonetheless, every morphological index was decreased with density, except for plant height under normal condition. Our field pot experiment results were also consistent with our previous prediction.

**Figure 6:**
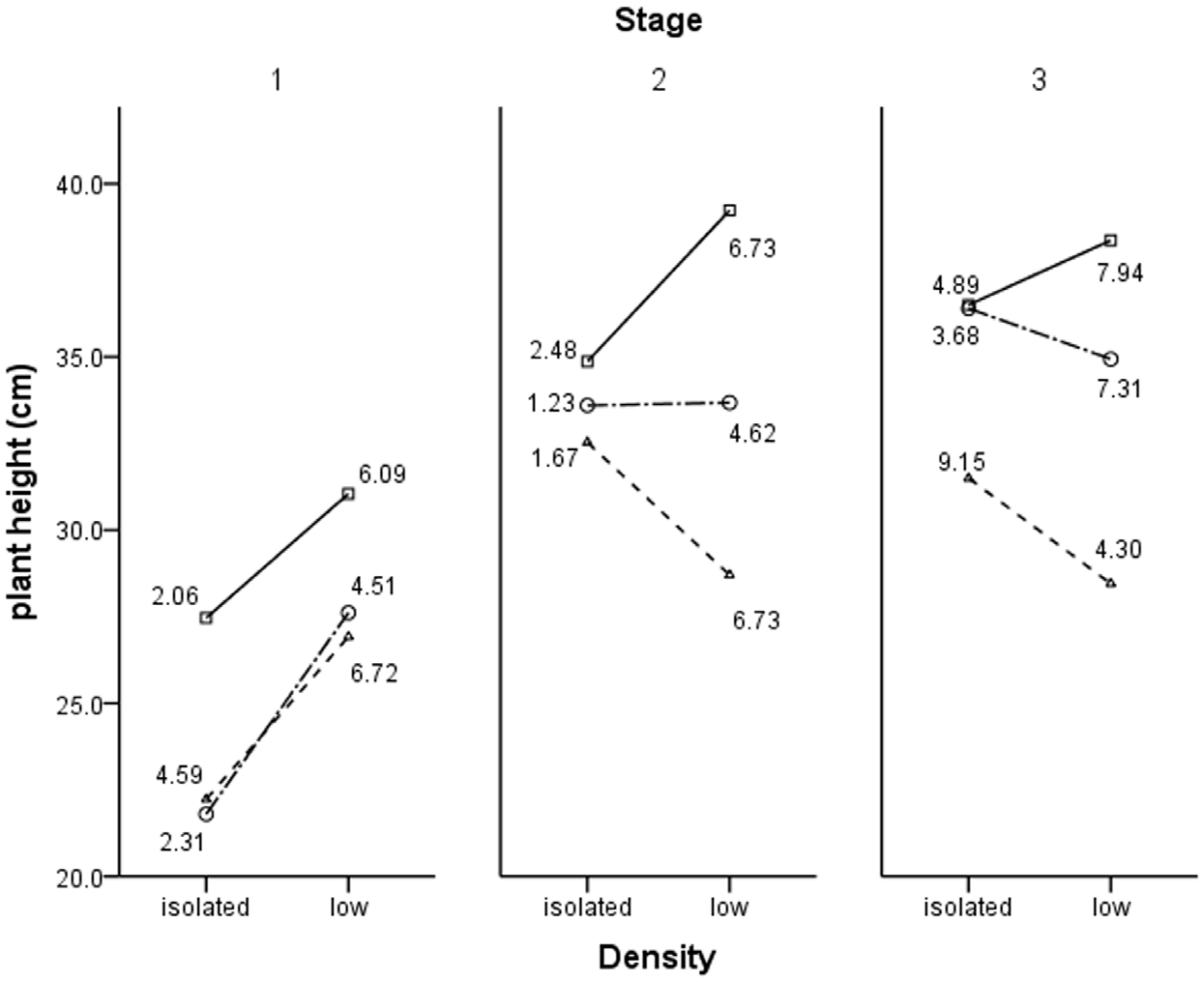
Height development of mung beans under different levels of density and UV-B radiation. The height of the isolated and low- density group plants was measured each month (branch number, leaf number, crown width, and leaf area per plant was also measured). Three symbols represent ambient conditions (-□-), low-enhanced UV-B radiation (-○-), and high-enhanced UV-B radiation (-△-). There were n = 9 plants for the isolated group (9 pots for each panel), but the morphology of five plants (low-density group) in the second month and eleven plants (low-density group) in the last month were unfortunately destroyed by an accident. Thus, there were n = 63 plants for low-density group in the first month (9 pots), n = 58 plants in the second month (9 pots), and n = 52 plants in the third month (9 pots). The number next to each point indicates the SD.

In terms of morphology, a trade-off has to be made between gaining light resources and averting the damage from the radiation as far as a sole individual is concerned because of the characteristic of UV-B irradiation. The trade-off was more complicated when an individual has a neighbor because of the additional trade-off between competition and positive interactions. This study focused on the four factors and two trade-offs. The competition and the desire for light resources resulted in plants choosing to enhance the height. However, the result of stress was actually the opposite. Trade-offs are also related to positive interactions because larger individuals obtain light resources more easily and also benefit from neighbors more greatly than smaller ones. Plants changed their strategy during various growing stages because of these trade-offs. The interactions among plants were not significant during the early development. Individuals tend to grow taller to obtain light and benefit under enhanced UV-B radiation. This condition was primarily because the desire for resources was more urgent for individuals in the early stage (Figure 6). However, the effects of stress exceed the influence of interactions and the desire for resources. Plant height then decreases with UV-B radiation in the following stages (Figure 6).

Trade-offs among stress, light resources, competition, and facilitation may affect the allocation strategy of plants corporately. Therefore, further research is needed in connection with these effects.

### Conclusion

The prediction that states the balance among stress, competition, and positive interactions collectively affect the self-thinning, size inequality, and phenotypic plasticity, is supported by the experimental results. Facilitation influences the pattern of the size structure of populations. More works in both mathematical and conceptual field should be conducted because the effects of facilitation and stress are difficult to separate in field experiment. Facilitation should not be neglected in the allocation of resources and self-thinning of individuals. Consequently, future studies should integrate facilitation in the current allometric theory mainly based upon competition. This integration would explicitly explain the allometric relationship of plants within populations.

## Materials and Methods

### Culture Conditions

Mung beans (*Vigna radiate*) cultivar Qindou-20 was grown in the biological garden of Northwest University (Xi’an, China, 34.3°N, 108.9°E; altitude 397 m). This species was chosen because of its short growth period and large leaves, which can effectively reflect the effects of radiation. The field pot experiment was designed to contain the three graded levels of density and three graded levels of supplemental UV-B radiation. Thus, there were a total of 3×3 treatments, with three replicates for each treatment. The growing season of mung beans lasts three months from May to July in the Xi’an region. Strong self-thinning was observed in the last month according to our preliminary experiment in 2009. Therefore, each month was chosen as a growing stage. We sampled 9 (treatments)×3 (replicates) pots of plants for measurements at the end of May, June, and July, respectively, so that 27×3 pots were prepared in the aggregate. The period of experiment followed the local farming of mung beans, which started from April 29, 2010 and finished at the end of July (86 days).

Pots were filled with soils obtained from the biological garden. Seeds of mung beans were then sown to the pots (diameter 25 cm, height 35 cm) with the following three densities: isolated (1 plant per pot), low-density (7 plants per pot), and high-density (37 plants per pot). The effects of irregular germination on the experiment were avoided by adding the prepared 27 pots of seedlings for the transplantation. These seedlings were separate from the 81 pots of mung beans. These 108 pots were then placed in the field. The distance between each pot was about 0.5 m and the plants were all under the same field management.

The mung bean seeds were sown in light of the hexagonal patterns. In this method, plants can use the space (resources) in an economical way; thus, different individuals can possess the same effective area [45]. Aside from the individuals on the edge of a pot, every plant was surrounded by six equidistant neighbors. Therefore, there were 7 individuals for the low-density group and 37 individuals for the high-density group to mimic the competition conditions and based on the characteristics of the hexagon. The distances between the two neighboring individuals were 1.5 cm and 3 cm for low- and high-density. Furthermore, 1 plant per pot was also prepared in the isolated group to distinguish the effects of UV-B radiation and the interactions among plants.

### UV-B Treatment

The first true leaf of each seedling would spring after one week or two. These mung beans seedlings were exposed to the following UV-B treatments:?ambient (as control), low-enhanced, and high-enhanced. Lamps were suspended above and perpendicular to the plants. The enhanced UV-B stress strength was expressed as the distance between the lamps and the top of the plants. In the low-enhanced and high-enhanced UV-B radiation groups, UV-B radiation source consisted of Qin brand (Baoji Lamp Factory, China) 40 W lamps. These lamps were filtered with one layer of 0.13 mm cellulose diacetate film that filters radiation below 280 nm [46], [47]. Spectral irradiance levels were measured by a UV radiometer (Instrument Company of Beijing Normal University, Beijing, China).

The UV-B_BE_ was estimated using the generalized plant response action spectrum normalized to 300 nm [48]. The daily enhanced UV-B radiation was set for 8 h per day (9∶00 to 17∶00). The lamp height above the plants was adjusted weekly to maintain a distance of 0.6 and 0.4 m between the lamps and the top of the plants. Supplemental irradiances of 3.60 and 5.62 kJ·m^2^ UV-B_BE_ were respectively provided. Based on calculation [48], these supplemental UV-B radiation levels were equal and can be increased at Xi’an, with 22% and 35% stratospheric ozone reductions during a clear day at the summer solstice (8.05 kJ·m^2^ UV-B_BE_ during clear sky conditions at the summer solstice). In the control group, plants only received ambient UV-B radiation. The UV-B lamps were also suspended, but were not provided with electric power to ensure that cultures can receive the same levels of sunlight with low- and high- enhanced radiation. Pots were rotated every 3 days or 4 days to minimize positional effects.

### Measurements and Statistical Analysis

The number of living individuals, stem height, basal diameter, crown width, and leaf areas were measured in the isolated and low-density group. In the pre-experiment, self-thinning occurred in the high-density group (only 10 to 20 survivals after self-thinning) instead of the low-density group (initial distance was 3 cm). As a result, strong interactions among plants in the high-density group were ensured by counting the number of living individuals and randomly selecting 10 individuals that own at least one neighbor not more than 3 cm away from them in each pot for the biomass measurements. Furthermore, only individuals in the middle were selected and those on fringe of the pot were not chosen to avoid the edge effect. Individuals under all treatments were then harvested and dried at 80°C until constant weight was achieved to collect stem, leaf, root, and fruit (pod) biomass of each plant. Only the total biomass was measured for the rest of the individuals in the high-density group (See Materials. S1).

Gaining light resources and avoiding UV-B stress is an important trade-off for an individual. When plants have neighbors there would be competition and positive interactions between them. This study focused mainly on the four factors and two trade-offs. However, in addition to competition, facilitation, desire for light resources, and stress, the high-density group introduced the fifth factor (self-thinning). Self-thinning influenced the plant morphology and provided difficulty in separating the effects of all the factors in the field pot experiment. As a result, the morphological indices of the high-density plots were ignored temporarily. This difficulty was the reason why different measurements were taken for the high-density treatment compared with the low-density treatment and the control.

Biomass was used to study size inequality because it reflects the sum of various effects on plants. The degree of size hierarchy for each treatment was estimated by comparing the Gini coefficient [49] and it is given by:
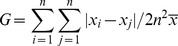
where *n* is the number of plants, 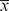 is the mean size, *i* = 1, *n* and *j* = 1, *n*, and x_i_, x_j_ are the sizes of *i*th and *j*th plants, respectively. The Gini coefficient ranges from 0 (all individuals have equal size) to a theoretical maximum of 1. The calculated G values were multiplied by n/(n-1) to obtain the unbiased values (G’) [50], [51]. Ninety-five percent confidence intervals for G’ were determined using the bootstrapping technique [52]. The 5000 bootstrapping samples were made in Matlab.

The Gini coefficient obtained from calculation utilized UV-B radiation and density as two fixed factors. The obtained data were evaluated statistically at the factorial level using two-way variance analysis (Two-way ANOVA). The significance was calculated at p<0.05 or p<0.001 level according to the Duncan’s multiple range test. Furthermore, the “n” reflects the number of plants in figure legends.

## Supporting Information

Table S1 F-values of two-way ANOVA for the effects of density, radiation, and their interaction on the Gini Coefficient of mung beans in every parameter (***P<0.001). The overall effects of UV-B radiation, density, and interactions (density×UV-B radiation) on the Gini Coefficient of mung beans were determined by the two-way analysis of variance (ANOVA). The significance was calculated at p<0.05 and at p<0.001 level based on Duncan’s multiple range test.
(DOC)

Materials S1 **Supporting materials show data of each biomass parameter (root, stem, leaves and fruit/pod size) under each UV-B radiation and density condition.**
(XLS)
