# Supplementary material for: Effects of Ultraviolet-B Irradiance on Intraspecific Competition and Facilitation of Plants: Self-Thinning, Size Inequality, and Phenotypic Plasticity
Source: PLoS One. 2012 Nov 30;7(11):e50822. doi: 10.1371/journal.pone.0050822 (PMC3511279; doi:10.1371/journal.pone.0050822)
Supplement: Table S1 — F-values of two-way ANOVA for the effects of density, radiation, and their interaction on the Gini Coefficient of mung beans in every parameter (***P<0.001). The overall effects of UV-B radiation, density, and interactions (density×UV-B radiation) on the Gini Coefficient of mung beans were determined by the two-way analysis of variance (ANOVA). The significance was calculated at p<0.05 and at p<0.001 level based on Duncan’s multiple range test. (DOC) [file pone.0050822.s001.doc]

| Parameter | Stage | Total df of the  Bootstrapping | F-value of Density | F-value of UV-B radiation | F-value of Density × UV-B radiation |
| --- | --- | --- | --- | --- | --- |
| Root | 1 | 30000 | 3007.454*** | 21980.474*** | 23029.391*** |
|  | 2 | 30000 | 787.4981*** | 17027.73*** | 574.0438*** |
|  | 3 | 30000 | 465.382*** | 6165.421*** | 5728.485*** |
| Stem | 1 | 30000 | 7931.323*** | 112.823*** | 2267.519*** |
|  | 2 | 30000 | 3985.469*** | 13586.62*** | 3725.974*** |
|  | 3 | 30000 | 9997.169*** | 12024.77*** | 1704.423*** |
| Leaves | 1 | 30000 | 19172.356*** | 18561.741*** | 7634.659*** |
|  | 3 | 30000 | 2340.1*** | 5293.339*** | 149.145*** |
|  | 2 | 30000 | 10995.232*** | 14643.066*** | 1983.476*** |
| Fruit | 2 | 30000 | 56372.782*** | 7119.48*** | 15694.242*** |
|  | 3 | 30000 | 457131.575*** | 6816.409*** | 71851.918*** |
| Total | 1 | 30000 | 4930.229*** | 3554.782*** | 40.242*** |
|  | 2 | 30000 | 19350.79*** | 12662.02*** | 9141.428*** |
|  | 3 | 30000 | 13808.102*** | 5570.622*** | 141.092*** |
